# Red Ginseng Extract Facilitates the Early Differentiation of Human Embryonic Stem Cells into Mesendoderm Lineage

**DOI:** 10.1155/2011/167376

**Published:** 2010-09-01

**Authors:** Yoon Young Kim, Seung-Yup Ku, Zev Rosenwaks, Hung Ching Liu, Sun Kyung Oh, Shin Yong Moon, Young Min Choi

**Affiliations:** ^1^Institute of Reproductive Medicine and Population, Medical Research Center, Seoul National University, Seoul 110-810, Republic of Korea; ^2^Department of Obstetrics and Gynecology, Seoul National University, College of Medicine, Seoul 110-510, Republic of Korea; ^3^CRMI, Cornell Medical College, New York, NY 10021, USA

## Abstract

Human embryonic stem cells (hESCs) have capacities to self-renew and differentiate into all cell types *in vitro*. Red ginseng (RG) is known to have a wide range of pharmacological effects *in vivo*; however, the reports on its effects on hESCs are few. In this paper, we tried to demonstrate the effects of RG on the proliferation and differentiation of hESCs. Undifferentiated hESCs, embryoid bodies (EBs), and hESC-derived cardiac progenitors (CPs) were treated with RG extract at 0.125, 0.25, and 0.5 mg/mL. After treatment of undifferentiated hESCs from day 2 to day 6 of culture, BrdU labeling showed that RG treatment increased the proliferation of hESCs, and the expression of Oct4 and Nanog was increased in RG-treated group. To find out the effects of RG on early differentiation stage cells, EBs were treated with RG extract for 10 days and attached for further differentiation. Immunostaining for three germ layer markers showed that RG treatment increased the expressions of Brachyury and HNF3*β* on EBs. Also, RG treatment increased the expression of Brachyury in early-stage and of Nkx2.5 in late-stage hESC-derived CPs. These results demonstrate facilitating effects of RG extract on the proliferation and early differentiation of hESC.

## 1. Introduction

Ginseng, the root of Panax Ginseng C. A. Meyer, has been widely used in oriental medicine for over a thousand years and is popular worldwide in alternative medicine [1, 2]. It has been known to possess a wide range of pharmacological effects including immunomodulatory, antiaging, anticancer, anti-inflammatory, antimutagenic activity and antioxidant activity [3–7]. In addition, ginseng is reported to have effects on various heart-related diseases and to have cardioprotective action [8].

Human embryonic stem cells (hESCs), which are derived from the inner cell mass of blastocyst, have capacities to self-renew indefinitely and differentiate into various types of cells *in vitro* [9, 10]. Therefore, hESCs are considered as a cell source for cell-based therapy and present an ideal model for *in vitro* drug screening and studies of human development. However, studies related to hESCs have focused on differentiation into specific cell lineage such as neurons [11], pancreatic *β* cell [12], cardiomyocytes [13], and the mechanism that regulates self-renewal or the signaling pathway for differentiation into certain cell types. Therefore, studies about the use of hESCs or derived differentiated cells as a developmental or preclinical model for the screening of molecules have been performed on a small, limited scale.

Recently, Wang et al. showed that treatment with the total saponins of Panax ginseng promoted the proliferation and differentiation into dopaminergic neurons of human embryonic neural stem cells [14]. Sasaki et al. demonstrated that treatment with a Panax ginseng compound promoted the differentiation of mouse embryonic stem cells into cardiomyocytes [15]. However, the effects of ginseng on the proliferation and differentiation of hESCs are still unknown.

In this study, we tried to demonstrate the effects of red ginseng extract on hESCs proliferation and early differentiation. Also, we analyzed the effects of red ginseng on the expression of cardiac related genes in hESC-derived cardiac progenitors (CPs).

## 2. Materials and Methods

### 2.1. Preparation of Red Ginseng Extract

Korean red ginseng extract derived from the root of *Panax ginseng* was provided from the Korean Ginseng Corporation (Seoul, Korea). Ginseng extract was dissolved in PBS (Invitrogen, USA) at concentration of 50 mg/ml and filtered through a 0.22 *μ*m filter (Millipore, USA). The dissolved extracts were aliquoted and stored at −20°C until use. Extracts were added to hESCs and differentiating cells with various concentrations—0.125, 0.25, and 0.5 mg/ml.

### 2.2. hESC Culture and EB Formation

The human embryonic stem cell line, SNUhES3 [10], was maintained as previously reported [16]. Briefly, hESCs were cultured on mitotically inactivated STO (CRL-1503; ATCC, USA) feeder layer by mechanical dissociation. hESC culture medium consisted of DMEM/F12 (Invitrogen), 20% knockout serum replacement (KO-SR; Invitrogen), 1% nonessential amino acids (Invitrogen), 50 U/ml penicillin (Invitrogen), 50 *μ*g/ml streptomycin (Invitrogen), 0.1 mM *β*-mercaptoethanol (Sigma, USA), and 4 ng/ml basic fibroblast growth factor (bFGF; Invitrogen).

For EB formation, undifferentiated hESCs were treated with Collagenase type IV (Invitrogen) for 30 min at 37°C. Dissociated hES colonies were centrifuged and washed with PBS. Washed hES colonies were transferred and cultured in suspension for several days.

### 2.3. Differentiation of hESC into Cardiac Progenitors

Undifferentiated hESCs were treated with 0.025% trypsin-EDTA (Invitrogen) for 3 min at 37°C. Feeder cells were gently removed and washed with PBS (Invitrogen). hES colonies were then treated with 100 ng/ml Activin A (R&D Systems, Minneapolis, MN, USA) for 24 hr and then 10 ng/ml BMP2 (R&D Systems) for another 4 days in RPMI 1640 (Invitrogen), supplemented with B27 (Invitrogen). The cytokines were removed at day 5 and medium was exchanged every other day during the differentiation period.

### 2.4. Measurement of hESC Colony Size

To calculate the average size of hESC colonies, clumps were replated in fresh feeder layer when subcultured. Four and six days after subculture, the diameters of 20 colonies were measured using *i* Solution Image Capture Program (Seoul, Korea).

### 2.5. BrdU Labeling Analysis

Cells were labeled with 10 *μ*M 5-bromo-2-deoxyuridine (BrdU, Roche Diagnostics, Germany) for 24 hrs to assess proliferation of RG-treated hESCs. After labeling, hESCs were detached from the feeder layer using 0.5 mg/ml dispase (Invitrogen) and detached colonies were treated with 0.25% trypsin-EDTA (Invitrogen) for 5 min at 37°C to dissociate into single cells. Single cells were fixed with 70% EtOH for 30 min at RT and denatured with 4 M HCl (Sigma) for 10 min at RT. Following incubation with 3% BSA soln. containing 0.03% Triton X100 (Sigma), cells were treated with mouse anti-BrdU antibody (Chemicon, USA) for 50 min at RT. After washing twice with PBST, the cells were incubated with Alexa Fluor 488-labeled donkey antimouse IgG antibody (Molecular Probes) for 40 min at 37°C, and then washed three times with PBST. Next, Propidium Iodide (Sigma) was added to the cells and incubated for 30 min at RT. Samples were analyzed using BD FACS Calibur (BD Sciences, USA).

### 2.6. RT-PCR and qPCR

Total RNAs were extracted from the cells using Trizol (Invitrogen) according to the manufacturer's instructions. cDNAs were synthesized from 1 *μ*g of RNA using Accute RT-premix (Bioneer, Korea). Primers used for RT-PCR and qPCR are listed in Table 1. PCR was performed by 30 cycles of denaturation at 95°C for 30 sec, annealing at 56°C for 30 sec and extension at 72°C for 30 sec. PCR products were visualized in 2% agarose gel.

Quantitative PCR was carried out in RotorGene 3000 (Corbett Life Science, Sydney, Australia) using QuantiTect SYBR green PCR kit (Qiagen, Valencia, CA, USA). The amplification program included an initial step at 95°C for 15 min, followed by 45 cycles of denaturation at 95°C for 15 sec, annealing at 58°C for 20 sec and extension at 72°C for 30 sec. All reactions were run in triplicate. C_T_ was calculated under default settings for the RotorGene 6.0 program (Corbett Life Science). Relative gene expression was normalized to GAPDH expression.

### 2.7. Immunofluorescence Staining

Samples were fixed with 4% paraformaldehyde (PFA; Sigma) for 30 min at RT. After washing with PBS, 3% BSA soln. containing 0.3% triton X100 (Sigma) was treated at 4°C overnight. Samples were then incubated with the primary antibodies, mouse antihuman Nestin (R&D Systems), rabbit antihuman Brachyury (SantaCruz Biotechnology, USA) and goat antihuman HNF3*β* (SantaCruz Biotechnology) for 1 hr at RT. After washing with PBST, incubations with the secondary antibodies, Alexa Fluor 488-labeled donkey antigoat and rabbit IgG, Alexa Fluor 594-labeled donkey antimouse IgG, were performed for 50 min at RT. Samples were washed with PBST, and mounting soln. containing DAPI (Vectorshield, USA) was added. Samples were observed using a confocal laser microscope (BioRad, USA).

## 3. Results

### 3.1. RG Extract Treatment Enhances Proliferation of hESCs

**Figure 1:**
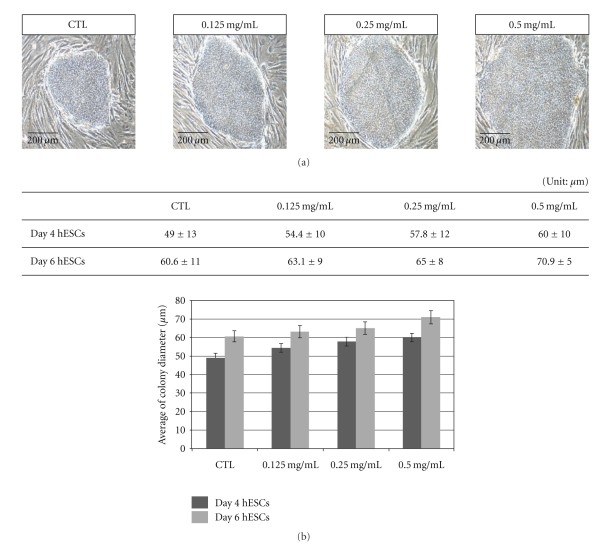
Morphological observation and measurement of colony diameter of undifferentiated hESCs after RG treatment. (a) hESCs were treated with 0, 0.125, 0.25, and 0.5 mg/ml of RG extract from day 2 to day 7. (b) Undifferentiated hESC colony diameter was calculated under the microscopic observation. Twenty colonies of each group were measured for the calculation of average diameter of hESC colony.

**Figure 2:**
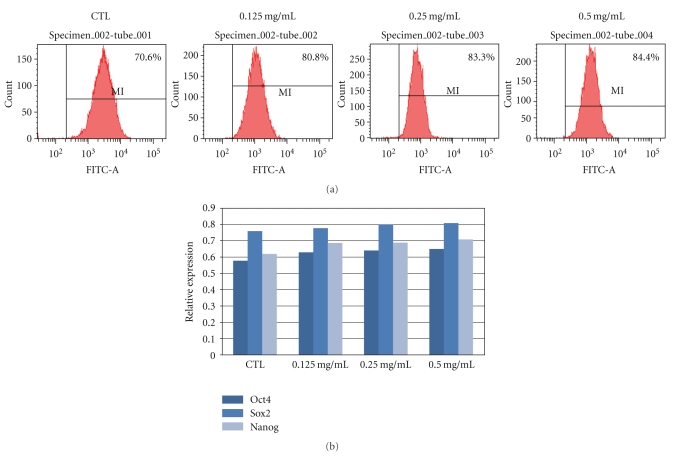
Proliferation and expression of pluripotency markers in RG extract-treated hESCs. (a) BrdU incorporation of RG-treated hESCs. Undifferentiated hESCs were preincubated with BrdU and analyzed by FACS. (b) Expression of stemness-related genes was evaluated by qPCR.

To analyze the effects of ginseng, undifferentiated hESCs were treated with 0.125, 0.25, and 0.50 mg/ml concentrations of RG extract from day 2 to day 6. RG-treated hESC colonies were enlarged in size compared to untreated hESCs (Figure 1(a)). Average diameters of both day 4 and day 6 hESCs increased in the RG-treated group compared to the control group (Figure 1(b)). To assess the proliferative effect, the BrdU-positive population was quantified. Compared to the control, there was an increase in proliferating cells in the RG-treated group. Proportion of positive cells were 80.8%, 83.3%, and 84.4% in 0.125, 0.25, and 0.5 mg/ml RG-treated groups, respectively (Figure 2(a)). These results showed that RG extract increased the proliferation of hESCs. 

To find out the effect of RG extract on pluripotency of hESCs, the expressions of Oct4, Sox2, and Nanog were evaluated. Undifferentiated hESCs generally proliferate and express high level of pluripotency markers. Expression of Oct4 was slightly increased in the RG-treated group. Expressions of Sox2 and Nanog were also increased in RG-treated group (Figure 2(b)). These results indicated that RG extracts also affected the expression level of pluripotency markers in undifferentiated hESCs.

### 3.2. Enhanced Differentiation into Mesendoderm at Embryoid Body Stage

**Figure 3:**
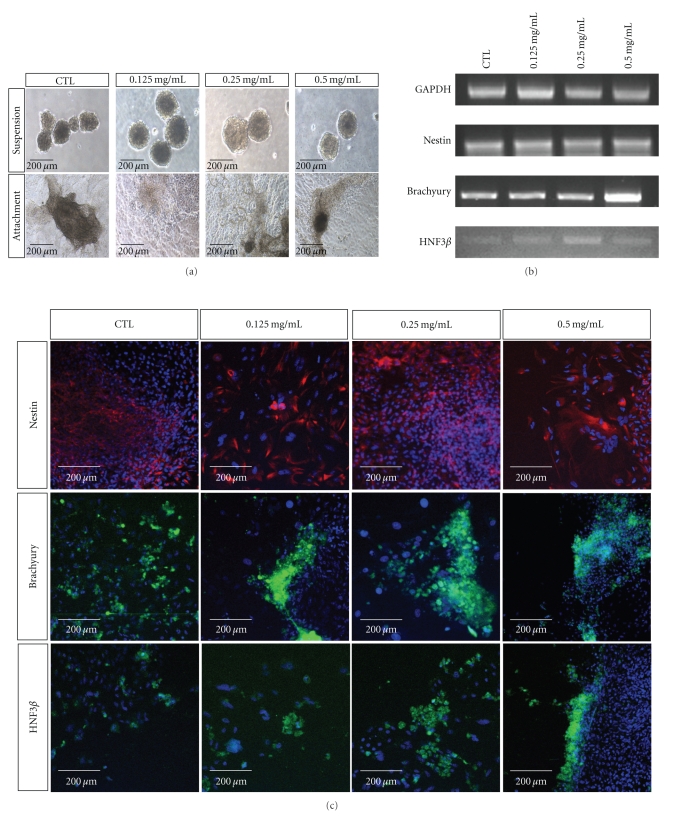
Expressions of three germ layer markers in RG-treated, differentiated cells. After 20 days of treatment, expression of early ectoderm, mesoderm, and endoderm markers were evaluated. RG-treated differentiated cells showed more dominant expression of mesodermal marker, Brachyury and endodermal marker, HNF3*β*. (a) Morphological observation of differentiating cells in suspension and attached state. (b) Expressions of three germ layer markers in reattached, RG-treated cells. (c) Immunostaining results of ectoderm (Nestin), mesoderm (Brachyury) and endoderm (HNF3*β*).

To evaluate the effects of RG extract on hESCs differentiation, RG extract was added to embryoid bodies in suspension culture for 10 days (Figure 3(a), upper panel). After 10 days of suspension, EBs were reattached for further differentiation (Figure 3(a), lower panel). RT-PCR results showed that expression of Brachyury and HNF3*β* was upregulated in the RG-treated group (Figure 3(b)). Immunostaining results also showed that RG treatment increased the expression of three germ layer markers, Nestin, Brachyury, and HNF3*β*, compared to the controls (Figure 3(c)). Interestingly, expressions of Brachyury and HNF3*β* were significantly upregulated in the RG-treated group. These results indicated that RG treatment of reattached EBs promoted the early phase differentiation of hESCs into mesendoderm lineage.

### 3.3. Upregulated Cardiac Lineage Gene Expressions in hESC-Derived CPs

**Figure 4:**
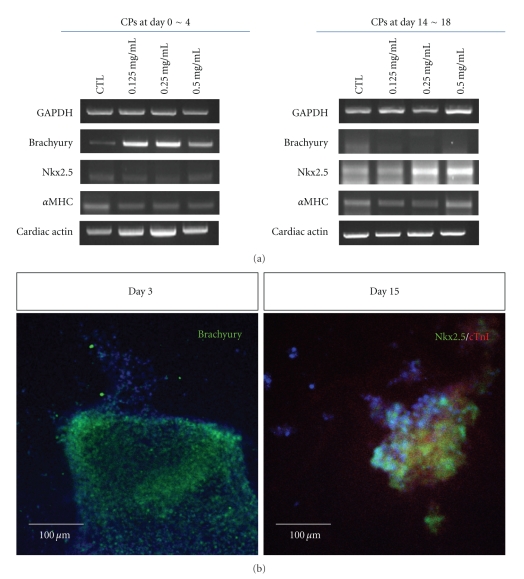
mRNA and protein expressions of mesodermal and cardiac markers in hESC-derived CPs. (a) mRNA expression of cardiac-specific markers in RG-treated, hESC-derived CPs. Expressions of Brachyury, Nkx2.5, *α*MHC, and cardiac actin were confirmed by RT-PCR. 1 *μ*g of RNAs were used for synthesis of cDNA. (b) Protein expression of Bachyury (early stage), Nkx2.5 and cardiac troponin I (late stage) in hESC-derived CPs.

Effects of RG extract on cardiac specific lineage differentiation were also evaluated. Differentiated cells of day 0 to 4 (early stage) and of day 14 to 18 (late stage) were treated with RG extract. Treatment with RG significantly enhanced the expression of Brachyury and of Nkx2.5 in early stage CPs (Figure 4(a)). Also, the expression of cardiac actin, another cardiac lineage specific marker, was also increased in RG-treated early stage CPs. At day 3 and day 15, the expressions of specific proteins were examined. Brachyury, Nkx2.5, and cardiac troponin (Tn) I showed increased expressions in the RG-treated group (Figure 4(b)). These results demonstrated that treatment with RG extract affected the expression of cardiac-related genes and promoted the differentiation of hESC-derived differentiated cells.

## 4. Discussion

Red ginseng is a popular herbal medicine with a long history of use in Asia and has a variety of therapeutic effects [17, 18]. The main component of ginseng is ginsenoside which belongs to the steroid family named dammarane saponins [17, 19]. Several studies have demonstrated the effect of ginseng on certain diseases using *in vitro* models. However, the evidence for the pharmacological effects of ginseng is still limited. Therefore, demonstrating the pharmaceutical activity of ginseng on a preclinical or clinical human model would be helpful in providing evidence for the effects of herbal medicine. 

The effect of red ginseng or ginseng extract on cell proliferation or differentiation has been previously demonstrated. Zuo et al. reported that total saponins of Panax Ginseng induced K562 cells to differentiate into erythrocytes [20], and Popovich et al. showed ginseng increased viability of cultured cell [21]. In addition, Lei et al. reported that the Panax ginseng extract promoted the migration of vascular endothelial cells and expression of vascular endothelial growth factor (VEGF) mRNA [22]. Another group demonstrated that treatment with ginseng extract enhanced the contractility of mouse embryonic stem cell-derived cardiomyocytes [15]. Even with these studies, in the scientific literature to date, the use of ginseng or red ginseng as gene expression-regulating factors in hESCs is extremely rare. To our knowledge, our current paper is the first to observe the facilitating effects of ginseng on the proliferation and differentiation of hESCs. This is important information since the aforementioned beneficial effects have been identified in human stem cells, which provides the basis for further studies to evaluate the long-term effects of RG.

hESCs usually proliferate unlimitedly *in vitro* without loss of pluripotency [9]. Many paracrine factors such as FGF2 contribute to the maintenance of stemness in hESCs [23–25]. Ginseng is known as a cardiotonic factor for cardiomyocytes [26] and regulator of cardiac contraction [27]. To determine the effects of RG extract, we observed the morphology after RG-treatment, based on the general tendency of hESCs to alternate their morphology after treatment with inducing or soluble factors. RG-treated hESCs showed larger colony size, increased in proliferation and expression of pluripotency markers. RG extract also promoted differentiation into mesendoderm lineage at embryoid body stage, which is evaluated by the increased expression of Brachyury and HNF3*β*. It may imply that ginseng extract could affect the cell differentiation at embryoid body stage. In addition, RG treatment increased the expression of Brachyury, an early mesendodermal marker, at the earlier stage of hESC-derived CPs and Nkx2.5, a cardiac-specific marker, at later stage of hESC-derived CPs. These results suggest that RG extracts have diverse effects according to the different stages of undifferentiated hESCs and differentiating cells.

**Figure 5:**
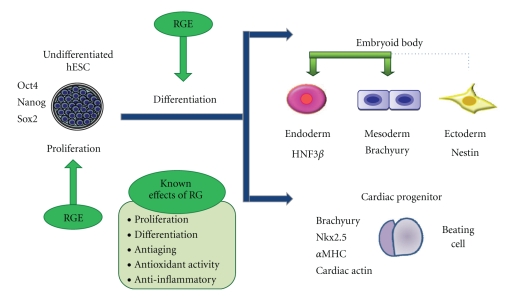
Schematic presentation on the various effects of red ginseng extract on the proliferation and differentiation of hESC, embryoid body and hESC-derived cardiac progenitor. hESC: human embryonic stem cells, RGE: red ginseng extract.

Intriguingly, we observed that different effects of RG were observed at the different stages of undifferentiated hESCs in differentiating cells. RG extract treatment led to an accelerated proliferation of undifferentiated hESCs, and promoted the differentiation of EBs into mesendoderm lineage (Figure 5). Further studies are needed to determine the effects of each RG component or its combinations, which may be able to explain the various effects of RG extract observed in our study.

In conclusion, this pilot study tried to evaluate the effects of RG extracts on the proliferation and differentiation of hESCs during short-term culture *in vitro*. From our results, RG extracts promoted the proliferation of undifferentiated hESCs, preferentially promoted differentiation of EBs into mesendoderm lineage, and upregulated cardiac-specific expression on hESC-derived cardiac progenitors.

## Figures and Tables

**Table 1:** Primer sequences used for RT-PCR and qPCR.

Gene	Forward	Reverse	bp
Pluripotency			

GAPDH	GGCGTTCTCTTTGGAAAGGTGTTC	GTACTCAGCGGCCAGCATCG	
Oct4	GAGAACAATGAGAACCTTCAGGA	CTCGAACCACATCCTTCTCT	
Sox2	GCGTACGCAAATTAAAGTCCAGA	TGCTATTCTTCGGCCAGTTG	
Nanog	GCGTACGCAAATTAAAGTCCAGA	TGCTATTCTTCGGCCAGTTG	

Differentiation			

Brachyury	TAAGGTGGATCTTCAGGTAGC	CATCTCATTGGTGAGCTCCCT	252
Cardiac actin	TCTATGAGGGCTACGCTTTG	CCTGACTGGAAGGTTAGATGG	630
HNF3*β*	CTACGCCAACATGAACTCCA	GAGGTCCATGATCCACTGGT	199
Nestin	CAGCTGGCGCACCTCAAGATG	AGGGAAGTTGGGCTCAGGACTGG	209
Nkx2.5	CTTCAAGCCAGAGGCCTACG	CCGCCTCTGTCTTCTTCAGC	233
*α*MHC	GTCATTGCTGAAACCGAGAATG	GCAAAGTACTGGATGACACGCT	413
